# A sodium channel mutant removes fast inactivation with the inactivation particle bound

**DOI:** 10.1085/jgp.202413667

**Published:** 2024-11-27

**Authors:** Yichen Liu, Francisco Bezanilla

**Affiliations:** 1 Department of Neurobiology, University of Chicago, Chicago, IL, USA; 2 Department of Biochemistry and Molecular Biology, University of Chicago, Chicago, IL, USA; 3 Centro Interdisciplinario de Neurociencias de Valparaiso, Valparaiso, Chile

## Abstract

Fast inactivation is a key feature of voltage-gated sodium channels and is pivotal for countless physiological functions. Despite the prevalence of the canonical ball-and-chain model, more recent structural results suggest that fast inactivation requires multiple conformational changes beyond the binding of the inactivation particle, the IFM motif. Combining ionic current, gating current, and fluorescent measurements, here we showed that a double mutant at the bottom of the pore domain (CW) removes fast inactivation by interrupting the communication of the IFM motif and the pore. Instead of triggering fast inactivation, the IFM motif binding in CW allows the channel to enter an alternative open state. This alternative open state severely influenced the voltage sensor movements and was not accessible to wild type or other fast inactivation–deficient channels. Our results highlight the multistep nature of the fast inactivation process in mammalian voltage-gated sodium channels and demonstrate that CW modifies the channel behaviors more profoundly than simple removal of fast inactivation.

## Teaser

In this work, Liu and Bezanilla describe a voltage-gated sodium channel mutant that removes fast inactivation while the fast inactivation particle, the IFM motif, remains bound. This work indicates that fast inactivation requires further conformational changes in addition to IFM motif binding.

## Introduction

Voltage-gated sodium (Na_v_) channels are the main drivers for membrane depolarization in excitation cycles (Hodgkin and Huxley 1952; Yu and Catterall, 2004). They operate in a classic positive feedback manner. Upon membrane depolarization, Na_v_ channels rapidly open, allowing sodium ion influx into the cell. This inward current in turn further depolarizes the membrane, driving more Na_v_ channels to open, generating the upstroke of the action potential. In this positive feedback, fast inactivation is pivotal to attenuate the inward sodium current and serve as the brake in this positive feedback loop. Since Hodgkin and Huxley, fast inactivation has been studied extensively and models have been put forward to explain the molecular mechanism for fast inactivation (Hodgkin and Huxley, 1952). The canonical “ball and chain” (Armstrong and Bezanilla, 1977) or the “hinged-lid” model (West et al., 1992) predict that the Na_v_ channel inactivation is the result of an intracellularly located inactivation particle, the isoleucine–phenylalanine–methionine (IFM) motif, binding to and subsequently blocking the open pore.

However, upon the resolution revolution of cryoEM and the numerous Na_v_ channels structures being solved, it became clear that the canonical model cannot account for the new experimental results. The structural results consistently show that in the inactivated state, the IFM motif, instead of blocking the open pore, is tucked away in a hydrophobic pocket distant from the pore (Huang et al., 2022; Jiang et al., 2020; Pan et al., 2018). This observation indicates that the IFM motif cannot serve as the inactivation gate that blocks the channel during inactivation. In our previous work, we demonstrated that the fast inactivation gate consisted of a two-layered hydrophobic barrier at the bottom of the pore, and the role of IFM motif seemed to be transducing the voltage-sensor domain (VSD) movements to the pore domain (PD) (Liu et al., 2023). One implication from this conclusion is that the fast inactivation process is more than a “two-state” model and likely involves multiple different conformational steps. Naturally, disruption in any of the conformational steps along the pathway would result in impaired fast inactivation.

In the literature, there exist numerous fast-inactivation–deficient Na_v_ channel mutants. Among them, the IQM mutant is probably the most studied (Kellenberger et al., 1997; West et al., 1992). In the IQM mutant, the phenylalanine in the IFM motif is mutated to a hydrophilic residue, glutamine (F1304Q, numbering based on rNa_v_1.4), which severely impedes the functioning of the IFM motif. As a result, IQM is able to remove most of the fast inactivation. However, due to the poor expression of the IQM mutant, an alternative mutant, the CW, has been utilized more and more as a model channel for fast-inactivation-deficient Na_v_ channel (Goldschen-Ohm et al., 2013; Wang et al., 2003). The CW mutant consists of two different mutations at the pore region of domain I (DI), L437C and A438W. While the mechanism of fast inactivation removal in IQM is well understood, exactly how CW inhibits fast inactivation remains elusive.

In this work, we set out to understand the mechanisms of the fast inactivation removal in CW. We found that CW and IQM behave differently in many ways, indicating that CW does not remove fast inactivation in a manner similar to IQM, namely by preventing the IFM motif binding. Experimentally, preventing the binding of the IFM motif in CW by applying ATX-II or utilizing the combined CW_IQM mutant reveals new phenotypes not seen before in CW, demonstrating the IFM motif still binds and influences the behaviors of the CW mutant. Our gating current and voltage clamp fluorimetry experiments demonstrate that the IFM motif binding seems to be driving the CW channel into an alternative open state that is not accessible in either wild type (WT) channels or IQM. In this alternative open state, VSD movements, particularly DII VSD, are greatly influenced. Based on our results, we demonstrate that CW removes fast inactivation not by impeding the IFM motif binding but rather by affecting the coupling between the IFM motif and the PD.

## Materials and methods

### Site-directed mutagenesis and in vitro cRNA synthesis

Rat Na_v_1.4 α and β1 subunit in pBSTA vector, flanked by β-globin sequences, were used in this study (Kubota et al., 2017; Liu et al., 2023). Mutagenesis was performed utilizing mismatch mutagenesis primers in a two-staged PCR reaction for best efficiency (Wang and Malcolm, 1999). The PCR products were first digested in DpnI (New England Biolabs) to remove the template and were then used to transform XL10-gold ultracompetent cells (Agilent). After ampicillin resistance screening, plasmids were purified using a standard DNA miniprep (Macherey-Nagel). Purified plasmids were then sent for whole plasmid sequencing for verification (Plasmidsaurus). Sequenced DNAs were linearized at the unique NotI-HF restriction site (New England Biolabs) and then transcribed into complementary RNA using T7 in vitro transcription kits (Ambion).

### *Xenopus laevis* oocytes preparation and heterologous channel expression

Ovaries of *Xenopus laevis* were purchased from XENOPUS1. The follicular membrane was digested by collagenase type II (Worthington Biochemical Corporation) at the concentration of 2 mg/ml and supplemented with bovine serum albumin (BSA, 1 mg/ml). After defolliculation, stage V–VI oocytes were then selected and microinjected with 50–150 ng of premixed cRNA with a 1:1 molar ratio of α and β1 subunits. Injected oocytes were incubated at 18°C for 1–5 days in SOS solution (in mM: 100 NaCl, 5 KCl, 2 CaCl_2_, 0.1 EDTA, and 10 HEPES at pH 7.4) supplemented with 50 µg/ml gentamycin prior to recording. Unless otherwise stated, all chemicals were purchased from Sigma-Aldrich.

### Cut-open voltage clamp and voltage clamp fluorometry

Ionic and gating currents in this study were recorded using the cut-open voltage-clamp technique, as previously described (Liu et al., 2023). Briefly, micropipettes filled with 3 M CsCl, with resistance between 0.4 and 0.8 MΩ, were used to measure the internal voltage of the oocytes. Current data were filtered at 20 kHz with a low-pass 4-pole Bessel filter online and sampled by a 16-bit AD converter at 1 MHz. All experiments were conducted at a low temperature of 10.5 ± 1°C unless otherwise stated, maintained by a feedback Peltier device. Ionic current experiments, unless otherwise stated, were conducted in an external solution consisting of, in mM, 28 Na methylsulfonate (MES), 92 N-methyl-D-glucamine (NMG) MES, 2 Ca MES, 10 HEPES, and 0.1 EDTA, pH = 7.4, and the internal solution consisted of, in mM, 12 Na MES, 108 NMG MES, 10 HEPES, and 2 EGTA, pH = 7.4. The low external sodium concentration was chosen to decrease the inward tail current, avoiding possible clamp issues. The capacitive transient was first compensated manually with a dedicated circuit and then further removed by an online P/-4 protocol (Armstrong et al., 1973) with a subtracting holding voltage of either −80 or −90 mV for ionic current experiments. For the ATX-II experiments, ATX-II obtained from Alomone Labs was added at 200 nM final concentration to the external solution. All the ATX-II solution was prepared fresh prior to the experiments. To facilitate the ATX-II binding, all the ATX-II experiments were performed at 18°C. Between experiments, 1% BSA in water was used to clean the chambers to minimize carrying over. For gating current experiments, all experiments were conducted in an external solution consisting of, in mM, 120 NMG MES, 2 Ca MES, 10 HEPES, and 0.1 EDTA, pH = 7.4, supplemented with 750 nM tetrodotoxin (TTX) and in an internal solution consisting of, in mM, 120 NMG MES, 10 HEPES, and 2 EGTA, pH = 7.4. A P/4 protocol with a subtracting holding voltage of 20 mV was used in this case for gating current. The speed of the voltage clamp, measured by capacitive transients, yields a time constant of around 75 µs.

Fluorescent measurements of VSD movements were carried out similarly as described before (Chanda and Bezanilla, 2002). Briefly, prior to tetramethyl rhodamine (TMR) labeling, oocytes were incubated in SOS solution supplemented with 0.1 mM DTT for 30 min. After washing three times in SOS solution, the oocytes were labeled in depolarizing solution (120 K MES, 2 Ca MES, 10 HEPES, 0.1 EDTA, pH = 7.4, in mM) with 10 µM TMR on ice for 30 min. Afterward, the oocytes were rinsed three times in SOS solution. Under voltage clamp conditions, the fluorophore was excited with a 530 nm LED (M530L3; Thorlabs). The filter cube consisted of a 535DF35 excitation filter, a 570DRLP dichroic mirror, and a 565EFLP emission filter (Chroma Technologies and Omega Optical). The emitted light was then focused onto a PIN-020A photodiode. The fluorescent signal was sampled by the same AD converter at 100 kHz with a 10 kHz online Bessel filter. The acquired fluorescent signal was then further processed offline and filtered at 1 kHz digitally.

### Data analysis

GraphPad 11 (Prism), Excel (Microsoft), Matlab R2023a (Mathworks), ChimeraX (Pettersen et al., 2021), and in-house software (Analysis and GPatchM) were used to process all the results.

(1) The ionic conductance (G(V_m_)) was calculated by dividing either the peak current or steady state current by the experimentally determined driving force at each depolarizing voltage (V_m_). Subsequently, the curve was normalized to the maximal conductance across all voltages (Gmax) to obtain the conductance versus voltage (GV) curves. The GV curves were fitted using a two-state model:

$$\frac{G\left(V_{m}\right)}{Gmax}=\frac{1}{1+e^{\left(V_{m}-V_{G\_1/2}\right)/k}}$$ (1)

where V_G_1/2_ is the half activation voltage and k is the slope.

(2) The time constant of gating current or ionic kinetics or fluorescent signals was calculated by fitting the traces with either one or two exponential decays using the general equation:

$$I\left(t\right)/F\left(t\right)=A_{1}e^{-\frac{t}{\tau_{1}}}+A_{2}e^{-\frac{t}{\tau_{2}}}+Iss/Fss$$ (2)

where A_1_, τ_1_, A_2_, and τ_2_ represent amplitudes and time constants of the first and second components, respectively. I_SS_ or F_SS_ is the steady-state current or fluorescence, respectively. When one exponential was used, the term of the second exponential component was eliminated.

(3) Total gating charge movement during voltage pulses was measured by integrating the gating currents. Charge–voltage (QV) curves were obtained by plotting the normalized total charge movement (Q_Norm_) at each depolarizing voltage and fitted using a two-state model:

$$Q_{Norm}\left(V_{m}\right)=\frac{1}{1+e^{\left(V_{m}-V_{Q\_1/2}\right)/k}}$$ (3)

where V_Q_1/2_ is the gating half activation voltage and k is the slope.

### Online supplemental material

**Figure S1.**
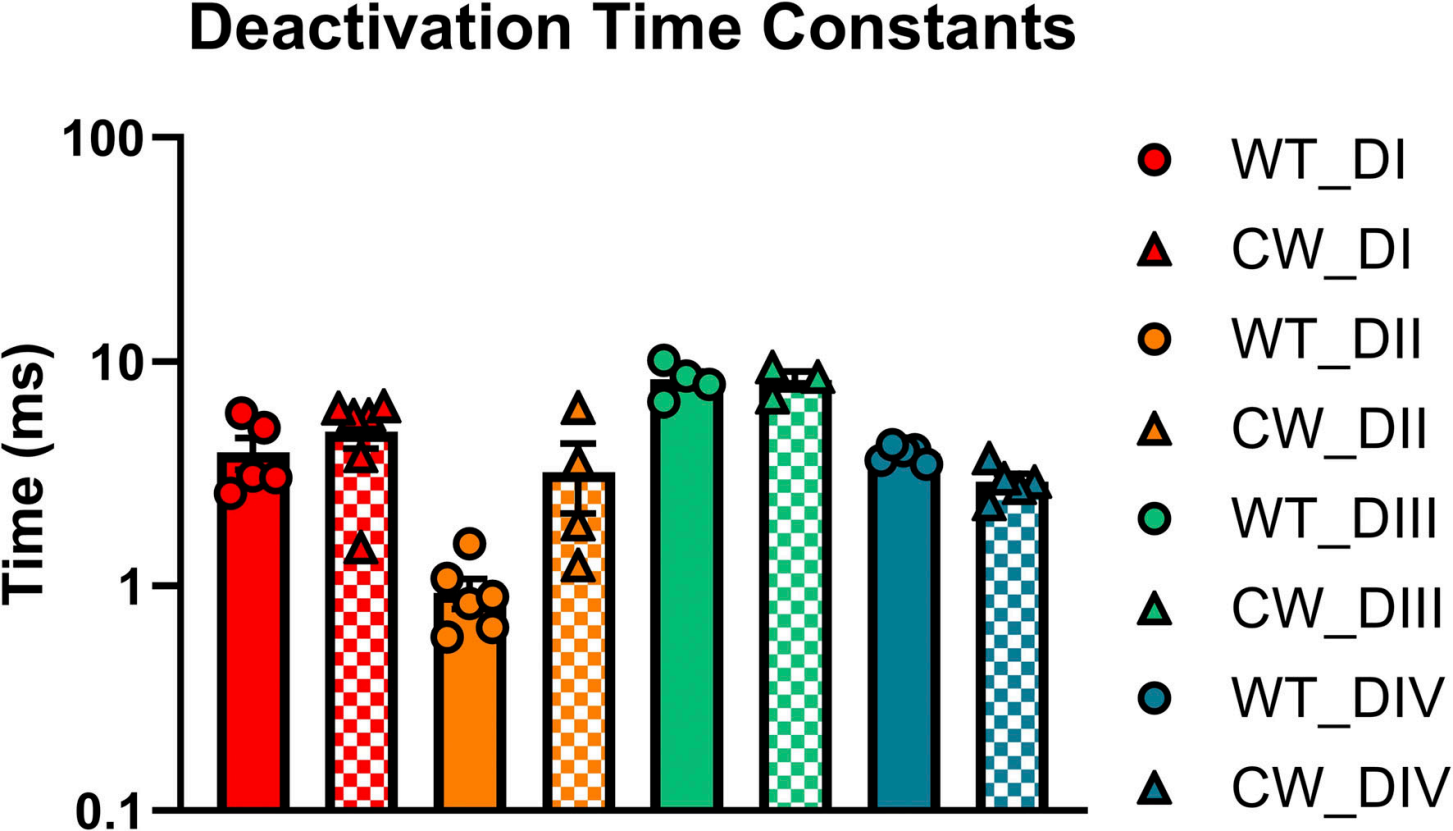
**Deactivation kinetics for all four voltage sensors in WT and CW.** The biggest difference in deactivation was seen in DII VSD.

Fig. S1 includes characterizations of the deactivation kinetics of the voltage sensor movements in WT and CW, highlighting the slowed return of DII VSD in CW to resting. Data S1 contains data underlying the figures.

## Results

### CW deactivates significantly slower and responds to site 3 toxin ATX-II differently compared with WT and IQM

**Figure 1.**
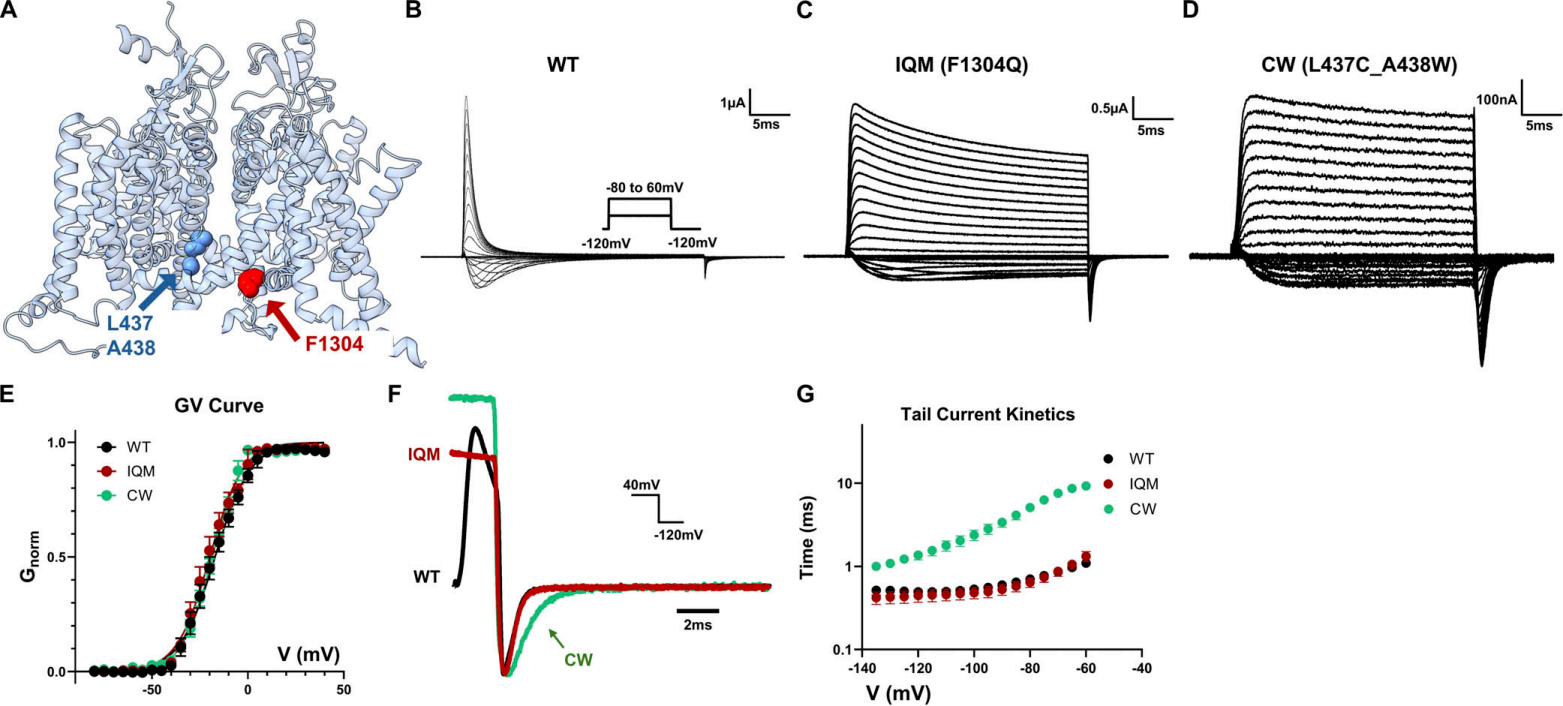
**GV curves and deactivation kinetics for WT, IQM, and CW. (A)** Structure of hNa_v_1.4 (PDB accession no. 6AGF) and the locations of the IQM mutation (F1304Q) and the CW mutation (L437C_A438W). **(B–D)** Example ionic current traces for WT, IQM, and CW, respectively. Both IQM and CW can remove most of the fast inactivation. The inset shows the voltage protocol. **(E)** GV curves for WT, IQM, and CW. All three constructs share similar GV curves. *N* = 6 for all constructs. Data plotted as Mean ± SEM. **(F)** Tail-current (deactivation) kinetics of WT, IQM, and CW after 2, 10, and 10 ms depolarization, respectively. The tail current was normalized to the peak. While WT and IQM share similar deactivation kinetics, CW has significantly slower channel closure. The inset shows the voltage protocol. **(G)** Fitted time constants for tail current were obtained after 2 ms depolarization for WT and 30 ms depolarization for IQM and CW. *N* = 6 for WT and IQM; *N* = 7 for CW. Data plotted as Mean ± SEM in log scale.

Even though an interaction between CW and the IFM motif is highly unlikely due to the distance in the structures (Fig. 1 A), as the initial step, we started by testing the hypothesis that CW removes fast inactivation by inhibiting the functioning of the IFM motif, similar to IQM. Then we would expect CW and IQM to share similar phenotypes. We recorded the ionic current from WT, IQM, and CW (Fig. 1, B–D). CW and IQM are similar in that both mutants could remove fast inactivation almost completely (Fig. 1, C and D) and share a similar voltage dependency of activation (GV curve, Fig. 1 E and Table 1). However, one key difference was seen in deactivation (Fig. 1 F). CW had significantly slower deactivation kinetics compared with the WT channels (Fig. 1 G). This slowed channel closure was not a result of the removal of fast inactivation since IQM and WT shared similar deactivation kinetics. Clearly, this observed slow deactivation is a unique phenotype of CW.

**Table 1.** Fit parameters of the GV curves for WT, IQM, CW, and CW_IQM used in Figs. 1 and 3

GV curve				
Mutants	WT	IQM	CW	CW_IQM
V50 (mV)	−17.63 ± 0.99	−20.27 ± 1.04	−18.44 ± 0.72	−35.34 ± 0.73
Slope (mV)	9.603 ± 0.82	8.959 ± 0.94	7.809 ± 0.631	8.227 ± 0.576
*N*	6	6	6	4

**Figure 2.**
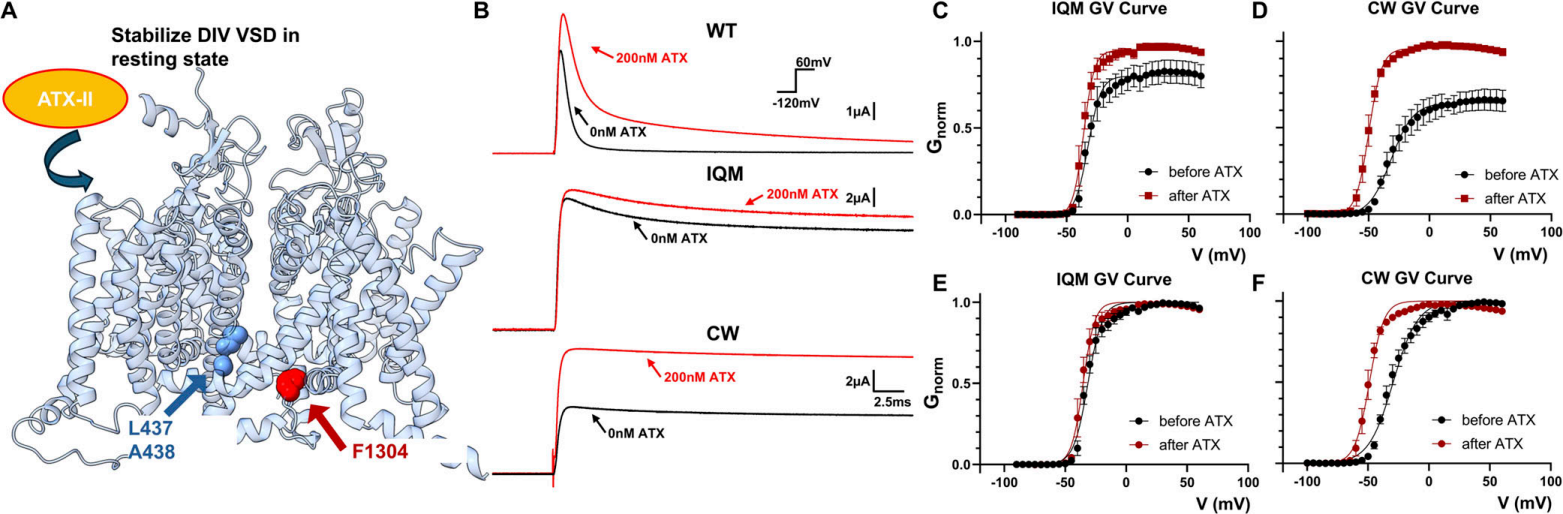
**Differential responses of ATX-II in WT, IQM, and CW. (A)** ATX-II is a site 3 toxin found in sea anemone. It can bind and stabilize the VSD in DIV in the resting conformation. **(B)** Example ionic current traces before and after the external application of 200 nM ATX-II. In WT, ATX-II slows down the fast inactivation kinetics as was demonstrated before. In IQM, minimal effect is observed. Around 10% increase in peak current is seen, likely due to the residual fast inactivation that is not removed. In CW, however, 200 nM ATX-II almost doubled the maximum current, an effect not seen in either WT or IQM. **(C and D)** GV curves for IQM and CW, respectively. The conductance is normalized to the maximum conductance after ATX-II treatment. **(E and F)** GV curves for IQM and CW, respectively. The conductance is normalized to the maximum conductance in each condition (with or without ATX-II). The GV curve of CW is shifted 15 mV to the left after ATX-II treatment and no significant change is observed in IQM. *N* = 4 for IQM and *N* = 5 for CW. Data plotted as Mean ± SEM. Data are fitted with a two-state model.

Next, we tested the effects of pharmacological agents on the mutant channels. Previous work has shown that local anesthesia, such as lidocaine and benzocaine, have similar binding affinity and efficacy on CW (Wang et al., 2004). However, when we tested the effect of ATX-II, CW showed a drastically different response. ATX-II is a site 3 toxin found in sea anemones that can bind and stabilize DIV VSD in the resting state (Fig. 2 A) (Richard Benzinger et al., 1999; Stevens et al., 2011). As a result, ATX-II slows fast inactivation in WT, increasing the peak current at the same time (Fig. 2 B, top panel). In IQM, since most of the fast inactivation has already been removed, ATX-II modification had little effect (Fig. 2 B middle panel, and C). In CW however, despite the apparent removal of fast inactivation, ATX-II modification led to almost doubling in the peak ionic current (Fig. 2 B bottom panel, and D) as if the channels became more conductive. Moreover, ATX-II treatment shifted the GV curve of CW around 15 mV to the left, a phenomenon not seen in IQM (Fig. 2, E and F; and Table 2). Given the key differences between IQM and CW, it is almost certain that CW utilizes different mechanisms to remove fast inactivation in Na_v_ channels and doesn’t work simply by impeding the functioning of the IFM motif.

**Table 2.** Fit parameters of IQM, CW and CW_IQM before and after ATX-II application used in Figs. 2 and 4

GV curve						
Mutants	IQM before ATX	IQM after ATX	CW before ATX	CW after ATX	CW_IQM before ATX	CW_IQM after ATX
V50 (mV)	−31.46 ± 0.84	−35.42 ± 0.83	−29.45 ± 0.98	−48.9 ± 0.76	−35.1 ± 0.85	−36.66 ± 0.82
Slope (mV)	5.835 ± 0.762	4.781 ± 0.738	10.06 ± 0.91	5.934 ± 0.69	8.062 ± 0.728	8.027 ± 0.701
*N*	4	4	5	5	4	4

### The IFM motif binding still happens in CW

**Figure 3.**
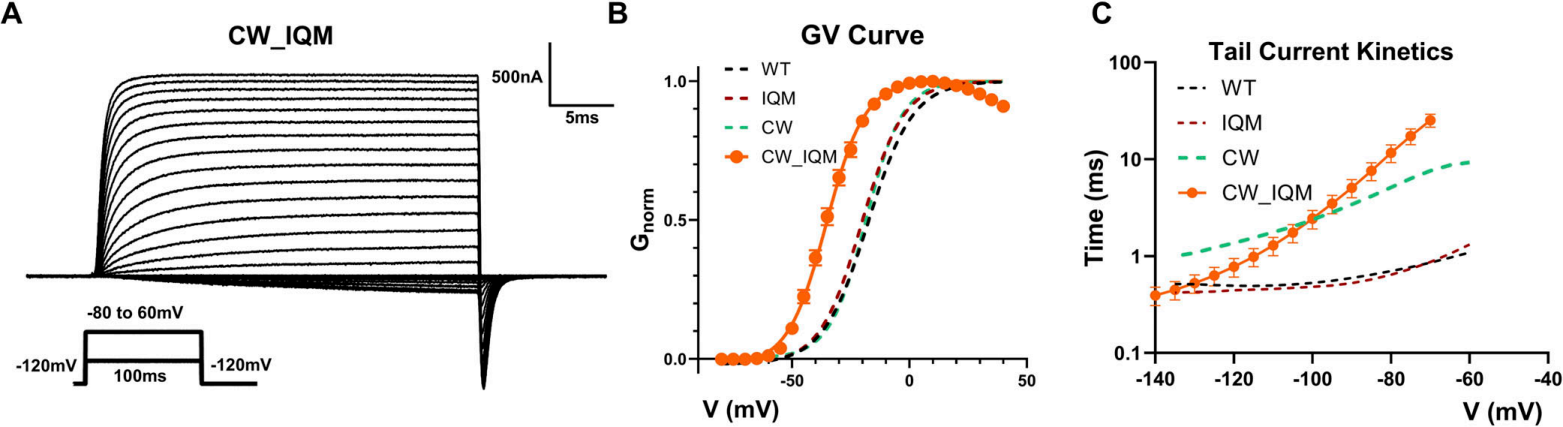
**Phenotypes of CW_IQM. (A)** Example ionic current traces for CW_IQM. Fast inactivation is completely removed. The inset shows voltage protocol. **(B)** GV curve for CW_IQM. Surprisingly, the voltage dependence of CW_IQM activation is significantly shifted to the left for around 15 mV compared with the WT, similar to the ATX-II treated CW. This shift in the GV curve is not observed in either IQM or CW, suggesting two mutations interact. *N* = 4. Data plotted as mean ± SEM. **(C)** Tail-current kinetics of CW_IQM compared with WT, IQM, and CW. *N* = 5.

If the CW and IQM don’t share the same mechanism, then it is possible that the IFM motif is still functioning in CW. To test this hypothesis, we combined CW and IQM, creating the CW_IQM construct (Fig. 3 A). By inhibiting the IFM binding in the background of CW, we significantly changed the channel behavior, shifting the GV curve almost 15 mV to the left (Fig. 3 B and Table 1). Additionally, channel closure was also modified by the addition of the IQM mutation to CW (Fig. 3 C). The emergence of these new phenotypes gives strong evidence that the IFM motif still binds in CW and by preventing its binding, the phenotypes of CW change.

### Binding of the IFM motif drives CW into an alternative open state

**Figure 4.**
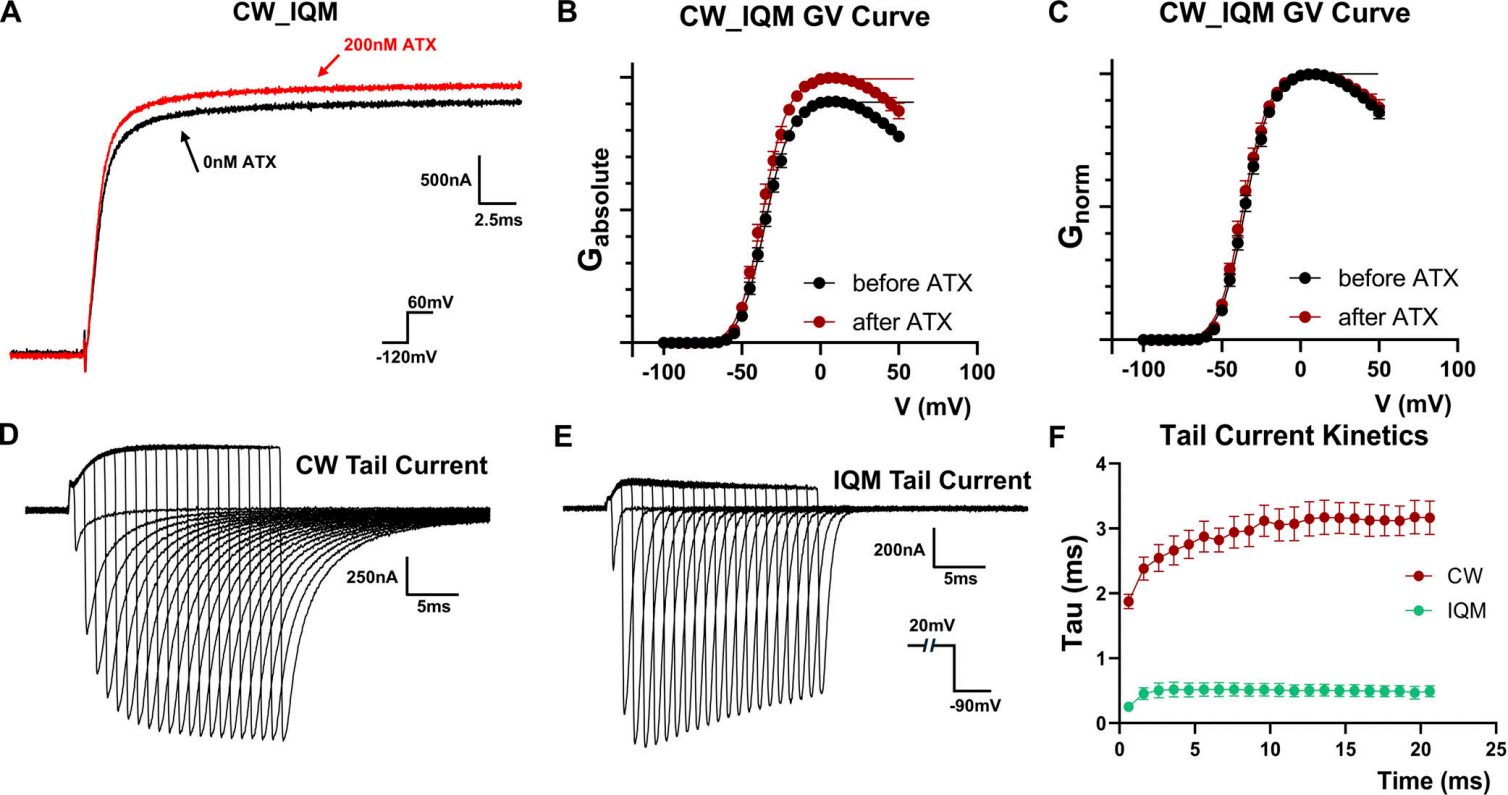
**Binding of the IFM motif drives CW into an alternative open state. (A)** ATX-II has minimal effect on CW_IQM unlike CW. Inset shows the voltage protocol. **(B and C)** GV curves for CW_IQM normalized to the maximum conductance after ATX-II treatment (B) or to the maximum conductance in each condition (C). *N* = 4. **(D and E)** Tail current with different depolarization times. Tail current was measured with difference depolarization time in (D) CW and (E) IQM. The inset shows the voltage protocol. **(F)** Tail current kinetics characterized as a function of depolarizing time. Note in CW, the tail current became slower and slower as depolarization time increases. Data plotted as Mean ± SEM. *N* = 5 for both cases.

Previous work has pointed out that DIV VSD is more involved in fast inactivation instead of activation (Chanda and Bezanilla, 2002; Capes et al., 2013). Thus, it is puzzling how in CW, the stabilization of DIV VSD in the resting state by ATX-II would lead to a left shift in the GV curve. The similarly shifted GV curve seen in CW_IQM raises the peculiar possibility that perhaps it is the binding of the IFM motif that altered the apparent GV curves. Therefore, we tested the effect of ATX-II on CW_IQM. We discovered that ATX-II stopped producing drastic modifications in CW_IQM (Fig. 4 A): no doubling in the ionic current (Fig. 4 B) nor further shift in the GV curve (Fig. 4 C and Table 2) was observed. It appears that ATX-II exerted its effect on CW not directly by impeding DIV VSD activation but rather by preventing the IFM motif binding.

The doubling in the peak ionic current seen in ATX-II-modified CW suggests that the IFM motif binding drives CW into an open state with low conductance and by inhibiting that transition, more ionic current could be released. To test the existence of multiple open states (Goldschen-Ohm et al., 2013), we investigated the effect of depolarization duration on deactivation kinetics in CW (Fig. 4 D). The deactivation kinetics in CW were becoming increasingly slower with longer depolarization time (Fig. 4 E), a strong piece of evidence for an alternative open state. Interestingly, similar behavior was not observed in IQM (Fig. 4, E and F). This is consistent with the hypothesis that in CW, the alternative open state is a result of the IFM motif binding and not due to the removal of fast inactivation.

### The alternative open state in CW is not accessible to WT nor IQM and severely impedes the VSDs transitioning into resting states

**Figure 5.**
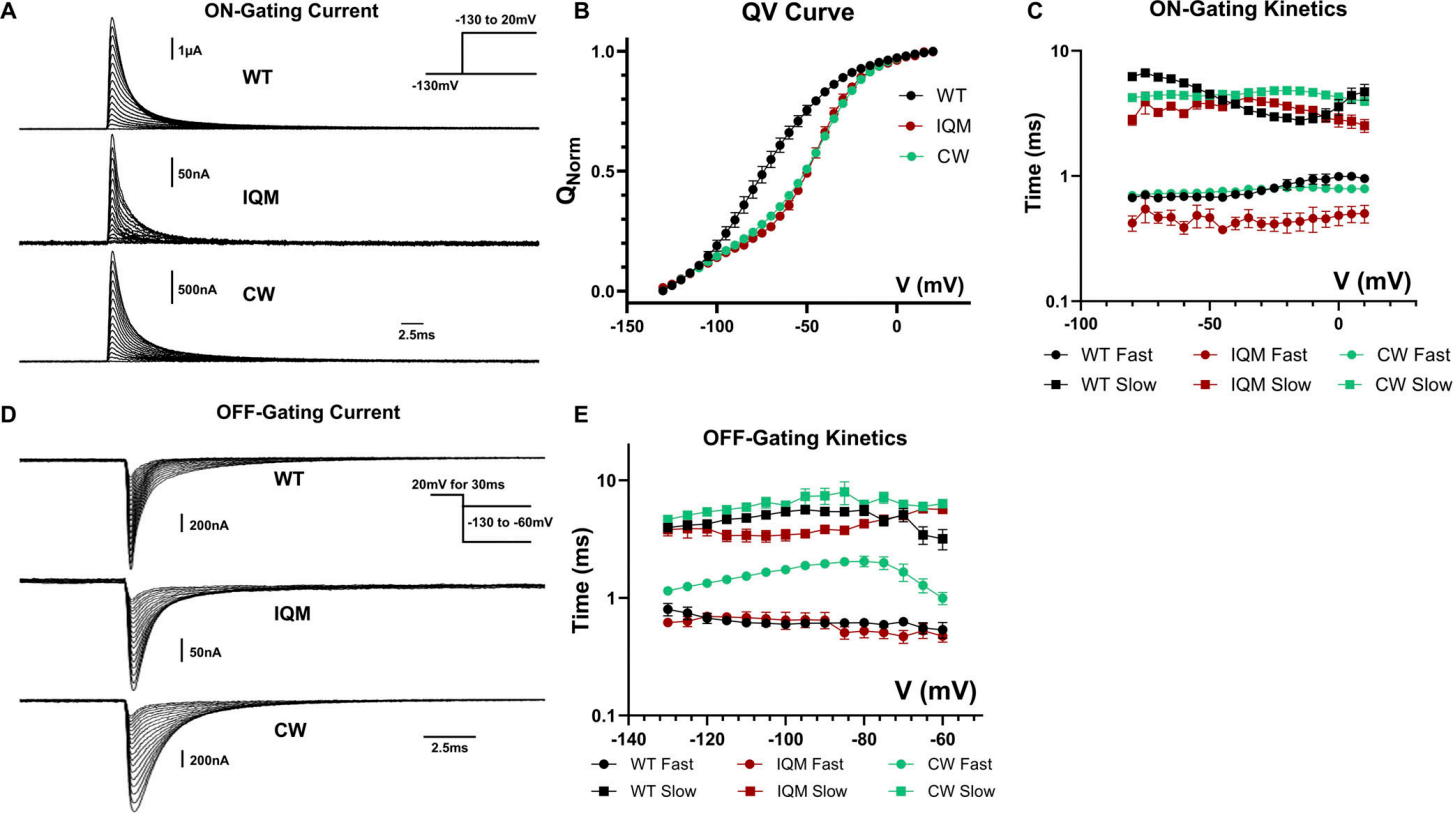
**Gating current for WT, IQM, and CW. (A)** Example on-gating current traces for WT, IQM, and CW. Inset show the voltage protocol. **(B)** QV curves for WT, IQM, and CW were obtained by integrating the off-gating current after 30 ms. Compared with WT, IQM, and CW both have a right-shifted QV curve. *N* = 6 for WT and CW, *N* = 5 for IQM. Data plotted as MEAN ± SEM. **(C)** On-gating current kinetics for WT, IQM, and CW. On-gating current was fitted with a two-component exponential function. **(D)** Example of off-gating current for WT, IQM, and CW. Inset shows the voltage protocol. **(E)** Off-gating kinetics fitted with two exponential decay components. Note that in CW, the fast component is significantly slower when compared with WT. *N* = 7 for WT, *N* = 6 for CW and *N* = 8 for IQM.

**Figure 6.**
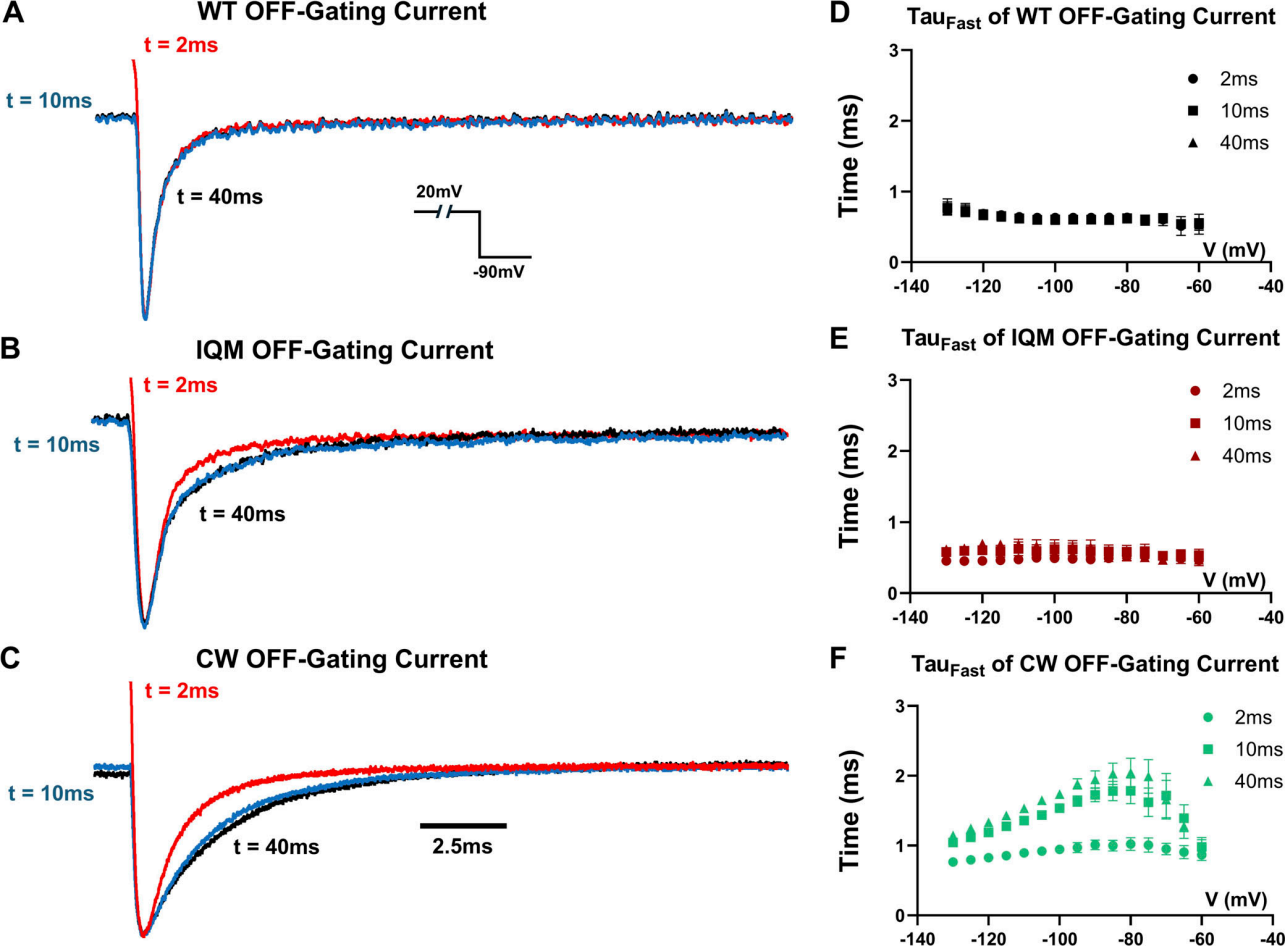
**Off-gating current kinetics for WT, IQM, and CW with different depolarization duration. (A–C)** Off-gating current from WT, IQM, and CW with 2 ms (red), 10 ms (blue), and 40 ms (black) depolarization. Note, only in CW, the fast component of off-gating current is getting slower. Inset shows the voltage protocol. Current is normalized to the peak. **(D–F)** fast time constant of WT, IQM, and CW characterized with different depolarization durations. In CW, the kinetics become significantly slower with longer depolarization. This is not seen in either WT or IQM. Data plotted are Mean ± SEM. *N* = 6 for WT, *N* = 7 for IQM, and *N* = 6 for CW.

Slower deactivation seen in the alternative open state of CW hints at the possibility of alteration to VSD movements. We recorded the gating current from WT, IQM, and CW (Fig. 5 A). A right shift in the voltage-dependent gating charge movement curve (QV curve) was observed in both IQM and CW (Fig. 5 B and Table 3). The on-gating current in CW shared similar kinetics as the WT (Fig. 5 C). However, significant changes in the off-gating current (Fig. 5 D) were observed. In CW, the fast component of the off-gating current was significantly slower than both WT and IQM. This observation was consistent with the slow deactivation kinetics in CW and is likely the underlying mechanism. Then, we utilized a similar voltage protocol to test whether the off-gating kinetics showed a similar dependency on depolarization duration (Fig. 6, A–C). In both WT and IQM, the fast components of the off-gating kinetics were insensitive to depolarization time (Fig. 6, D and E). However, in CW, the fast component became slower and slower as depolarization continued (Fig. 6 F), similar to its tail current kinetics, another unique behavior of CW.

**Table 3.** Fit parameters of the QV curves for WT, CW and IQM used in Fig. 5

QV curve			
Mutants	WT	CW	IQM
V50 (mV)	−77 ± 2.41	−49.55 ± 1.24	−48.31 ± 1.31
Slope (mV)	22.11 ± 2.22	21.54 ± 1.37	17.23 ± 1.52
*N*	6	6	5

Our gating current experiments demonstrated that the slowed deactivation seen in CW most likely originated from the slowed movement of the VSDs. As CW progressed into the alternative open state, the VSD movements became increasingly slower. Notably, this unique time-dependent slowing down of VSD movements was only observed in CW. A similar phenomenon was not observed in either in WT or in IQM. This indicates that the alternative open state seen in CW is not revealed by the removal of fast inactivation and is not accessible in normal channels.

### The slowed off-gating current in CW was mostly due to altered DII VSD movements

**Figure 7.**
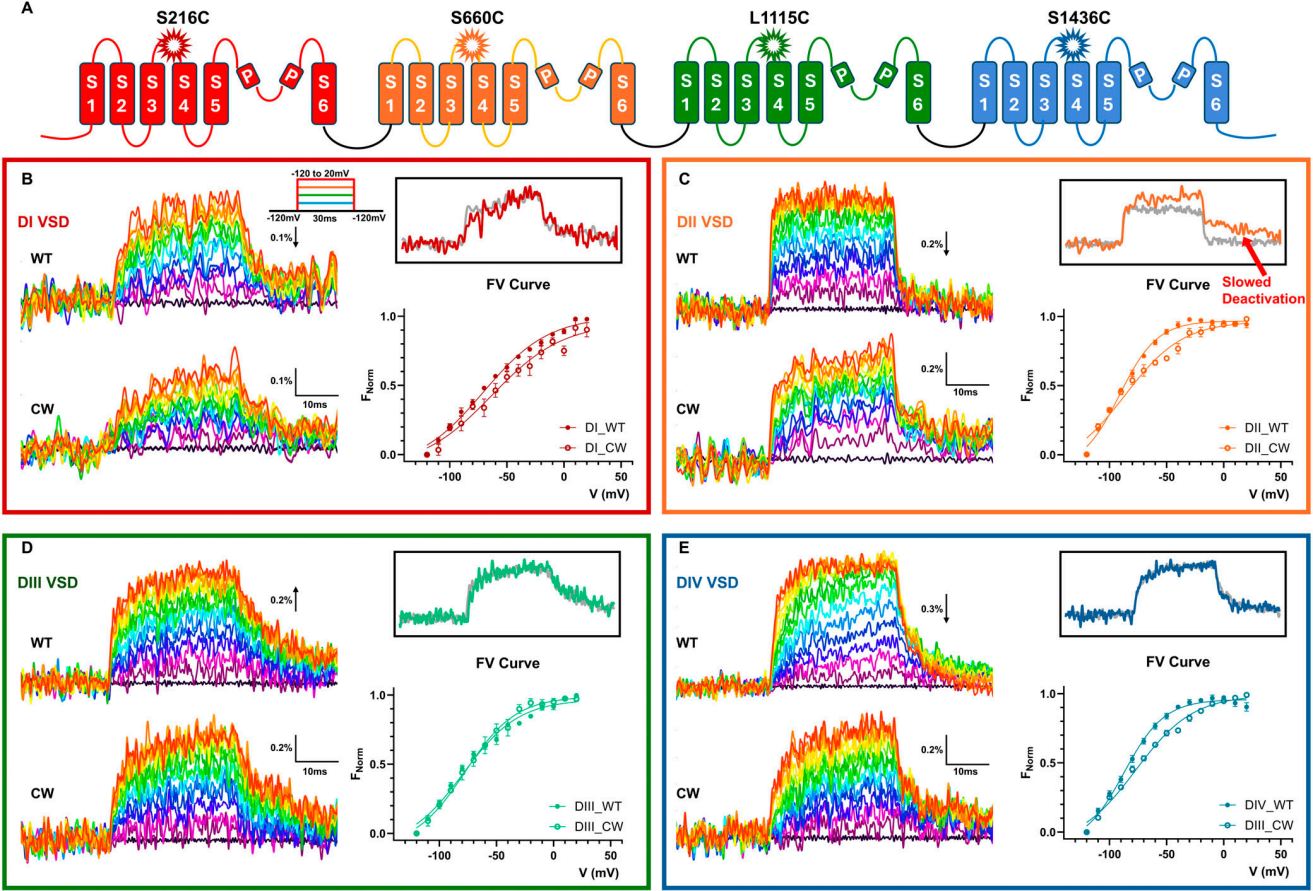
**Individual VSD movements are tracked by voltage clamp fluorometry. (A)** Diagrams showing the previously identified locations used for TMR labeling in all four VSDs. **(B–E)** Fluorescent signals from DI (B), DII (C), DIII (D), and DIV (E). Signals were filtered at 1 kHz offline. Insets with black boundaries show the normalized fluorescent signal comparison of WT (gray) and CW (colored) at the most depolarized voltage tested (20 mV). Note in C, DII VSD deactivation showed a significant slow component that was not present in WT. Data plotted as Mean ± SEM. Amplitude measured as ∆F/F. The direction of the arrow indicts direction of fluorescent changes.

To pinpoint the origin of the slow off-gating recorded from CW, we performed site-directed fluorometry experiments to monitor each individual VSD movement (Fig. 7 A). Due to the low expression level of IQM, fluorescent signals could not be reliably resolved and therefore, only WT and CW will be compared here. Voltage-dependent fluorescent signals (FV) were measured at the steady-state level. In CW, DI, DII, and DIV showed a slight right shift in the FV compared with the WT (Fig. 7, B, C, and E; and Table 4), consistent with the gating current experiments. VSD movements in DIII seem not to be significantly influenced in CW (Fig. 7 D and Table 4, inset). In DII, in addition to the shift in the FV curve, a slow component appeared as the VSD was transitioning into the resting state (Fig. S1). It was shown before that the fast component of the off-gating current originated mostly from the VSD movement in DI and DII (Cha et al., 1999; Chanda and Bezanilla, 2002). This slow return to the resting state in DII VSD most likely contributed to the slowed off-gating current observed in CW.

**Table 4.** Fit parameters of the FV curves for WT and CW used in Fig. 7

FV curve		
DI mutants	WT	CW
V50 (mV)	−70.77 ± 3.21	−63.34 ± 8.2
Slope (mV)	28.76 ± 2.63	31.2 ± 6.07
*N*	5	5
DII mutants	WT	CW
V50 (mV)	−91.121.24	−86.18 ± 3.68
Slope (mV)	16.52 ± 1.1	25.23 ± 3.25
*N*	6	6
DIII mutants	WT	CW
V50 (mV)	−79.03 ± 2.93	−77.43 ± 3.62
Slope (mV)	24.34 ± 2.65	22.22 ± 2.85
*N*	4	3
DIV mutants	WT	CW
V50 (mV)	−87.27 ± 1.62	−77.21 ± 2.34
Slope (mV)	17.67 ± 1.4	25.22 ± 1.93
*N*	6	7

## Discussion

### Molecular mechanism of fast inactivation removal in CW and fast inactivation deficient Na_v_ channel model

CW consists of two point mutations at the PD of DI, L437C, and A438W. In this work, we demonstrated that the DIII–DIV linker movement was not impaired by these mutations, and it seems to be the downstream coupling that is influenced. One plausible mechanism is that the bulky side chain introduced by the tryptophan mutation at the 438 position doesn’t allow the conformational transition from an open pore to an inactivated pore. Attempting to accommodate the conformational changes induced by the IFM motif, instead of entering into the fast inactivated state, CW is driven into an alternative open state that appears to have significantly small conductance and is inaccessible to the normal channels. Even though the exact molecular mechanism would certainly require further investigation, it seems hard to avoid the conclusion that CW doesn’t serve as a good model to study the Na_v_ channels in the absence of fast inactivation. Our gating current, fluorescent, toxin, and deactivation experiments all consistently point out the significant deviations of CW behavior from WT and IQM. Without the rightful comparison with IQM, the canonical model for fast inactivation deficient Na_v_ channel, one could easily reach the wrong conclusion that all the behaviors seen in CW are results of fast inactivation removal. For the time being, despite its poor expression level, IQM still seems to be the best option to study Na_v_ channels in the absence of fast inactivation.

### Influence of pore mutations on VSD movements

In CW, the deactivation kinetics is significantly slower than that of WT and IQM. Our voltage-clamp fluorometry experiments suggested that this decrease in deactivation speed is likely due to slowed movement of DII VSD. This is also consistent with the observation from the gating current experiments where the fast component of the gating current became increasingly slower with longer depolarization. Although we failed to record reliable fluorescent signals from the IQM mutant, results from Jonathan Silva’s group on Na_v_1.5 in the IQM mutant showed that DII VSD movement was not significantly modified (Hsu et al., 2017); no slow component was observed in the DII VSD deactivation. This provides further evidence that this phenotype is unique to CW and doesn’t reflect the behaviors of Na_v_ channels in the absence of inactivation. Mechanistically, it is possible that the pore conformation in the alternative open state introduced by CW partially stabilizes the DII VSD or even the immobilized DII VSD. As a result, the return of the gating charges in DII VSD becomes very slow.

In addition to DII, VSDs from DI and DIV also seemed to be modified. Small right shifts of the FV curves can be resolved in DI and DIV VSDs, which could explain similar shifts observed in the QV curves. It seems that CW modifies the VSD movement in all domains except DIII. It is possible that the influence of VSD movements is exerted through the S6 helices where the mutations reside. DI S6 is in close contact with both DII and DIV S6, which in turn, communicate with the voltage sensors via the S4–S5 linkers.

### Multiple open states in Na_v_ channels

In this work, we investigated the mechanism of fast inactivation removal in the CW mutant. We found that in CW, the IFM motif binding drives the channel into an alternative open state. Multiple open states have been postulated and observed in Na_v_ channels before (Capes et al., 2013; Chanda and Bezanilla, 2002; Correa and Bezanilla, 1994; Goldschen-Ohm et al., 2013) and most of them are associated with the DIV VSD movement contributing to an alternative pore conformation. A clear distinction needs to be made here. Even though in CW, the entry into this alternative open state follows closely the DIV VSD movement as demonstrated by previous work (Goldschen-Ohm et al., 2013; Lewis and Raman, 2013) and our ATX-II experiments, in CW, it is the binding of the IFM motif that seems to trigger the entering into this state. The CW_IQM construct inhibits the function of the IFM motif and completely negates the effect of ATX-II, which highlights the importance of IFM binding to the efficacy of this site III toxin. Since the movement of the IFM motif itself follows closely the DIV VSD movement (Capes et al., 2013), it should be no surprise that previous single-channel work has reported the DIV VSD movements following the appearance of the alternative open state (Goldschen-Ohm et al., 2013). It is important to point out that this alternative open state observed in CW seems to be only accessible to CW and cannot be entered by WT or other fast inactivation deficient mutants (IQM). Only in CW, did we observe (1) significant modification of ATX-II, (2) time-dependent slowing down of deactivation, and (3) time-dependent slowing of off-gating current. Most likely, the observed open state in CW is a new state created by the mutation itself and is not present in normal channels. However, this is not to claim that more than one open state doesn’t exist in Na_v_ channels; in fact, a third open state has been reported in CW (Goldschen-Ohm et al., 2013).

### Coupling between IFM motif binding and channel activation

One puzzling finding from the current results comes from the observed changes in GV curves due to IFM motif bindings. Even though it is long established that activation and fast inactivation are coupled in Na_v_ channels (Armstrong et al., 1973), it is unclear how in CW, inhibition of IFM binding, likely an early step in fast inactivation, influences back the activation. Given the significant influence of CW on VSD movements in almost all VSDs, it is possible that independent from the alternative open state, CW already influences the channel behaviors in the canonical open state. The tail current kinetics seems to support this hypothesis in that even with short depolarizations, the deactivation kinetics was never quite as fast as the WT or IQM. And, in CW_IQM, where the entry into the alternative open state is presumably mostly inhibited, the deactivation kinetics differed from WT and IQM, which suggests modifications to the canonical open state in CW.

### Fast inactivation as a multistep process in Na_v_ channels

**Figure 8.**
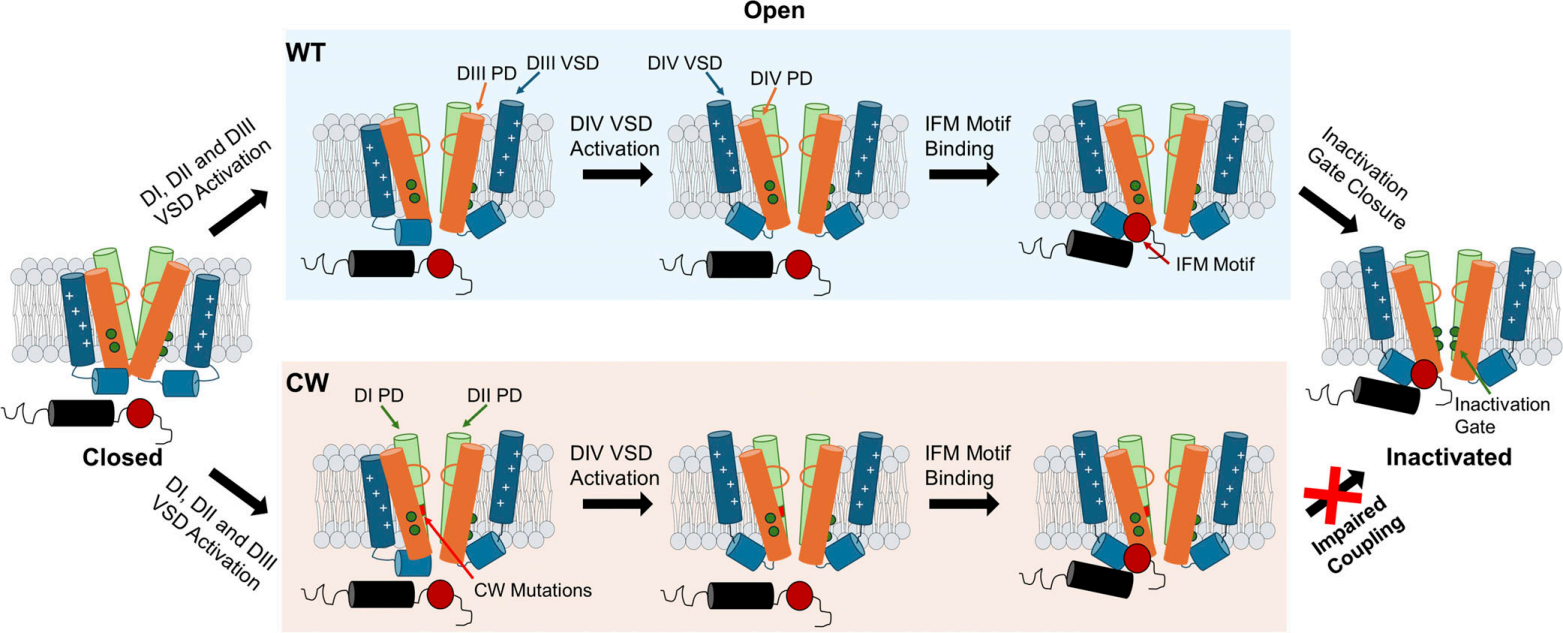
**Cartoon representations of fast inactivation process in** **WT** **Na_v_ channel and CW mutant.** Voltage sensors in DI and DII are omitted for simplicity’s sake. Upon the onset of depolarization, VSDs from DI to DIII activate and open the channel. Later DIV VSD activates, allowing the binding of the IFM motif. The binding of the IFM motif in turn triggers a conformational change at the pore, which drives the closure of the inactivation gate at the bottom of the S6 helices. In the case of CW, however, all the conformational changes happen until the IFM motif binding. Due to the mutations, the conformational changes triggered by the IFM motif binding are impaired and subsequently in CW, the channels do not enter the fast inactivated state.

Our CW_IQM and ATX-II experiments demonstrated that in CW, a mutant Na_v_ channel that doesn’t fast inactivate, the IFM motif is still bound. This conclusion is consistent with our previous work where we proposed that the fast inactivation gate is located at the bottom of the pore in S6 (Liu et al., 2023) and that fast inactivation is a multistep process. From the current point of view, this process at least involves two distinct steps (Fig. 8). First, the IFM motif is required to bind to its binding pocket. The binding of the IFM motif itself doesn’t lead directly to fast inactivation, but rather it triggers further conformational changes, most likely in DIII and DIV S6 helices. This conformational change allows the double-layered hydrophobic ring to block the ion permeation and drive the channels in the fast inactivated state. In CW, the conformational changes triggered by IFM motif binding seem to be significantly altered; instead of entering the fast inactivated state, the channels enter an alternative open state. As a result, CW removes the fast inactivation with the inactivation particle still bound.

## Supplementary Material

Data S1 contains data underlying the figures.

## Data Availability

The data underlying this study are available from the corresponding author upon reasonable request.
